# Iodine and Mental Development of Children 5 Years Old and Under: A Systematic Review and Meta-Analysis

**DOI:** 10.3390/nu5041384

**Published:** 2013-04-22

**Authors:** Karim Bougma, Frances E. Aboud, Kimberly B. Harding, Grace S. Marquis

**Affiliations:** 1 School of Dietetics and Human Nutrition, McGill University, 21111 Lakeshore Road, CINE Building, Sainte Anne-de-Bellevue, QC, H9X 3V9, Canada; E-Mail: karim.bougma@mail.mcgill.ca; 2 Department of Psychology, McGill University, 1205 Dr. Penfield Avenue, Montreal, QC, H3A 1B1, Canada; E-Mail: frances.aboud@mcgill.ca; 3 Micronutrient Initiative, 180 Elgin Street, Suite 1000, Ottawa, ON, K2P 2K3, Canada; E-Mail: kharding@micronutrient.org

**Keywords:** children, iodine, mental development, systematic review, meta-analysis

## Abstract

Several reviews and meta-analyses have examined the effects of iodine on mental development. None focused on young children, so they were incomplete in summarizing the effects on this important age group. The current systematic review therefore examined the relationship between iodine and mental development of children 5 years old and under. A systematic review of articles using Medline (1980–November 2011) was carried out. We organized studies according to four designs: (1) randomized controlled trial with iodine supplementation of mothers; (2) non-randomized trial with iodine supplementation of mothers and/or infants; (3) prospective cohort study stratified by pregnant women’s iodine status; (4) prospective cohort study stratified by newborn iodine status. Average effect sizes for these four designs were 0.68 (2 RCT studies), 0.46 (8 non-RCT studies), 0.52 (9 cohort stratified by mothers’ iodine status), and 0.54 (4 cohort stratified by infants’ iodine status). This translates into 6.9 to 10.2 IQ points lower in iodine deficient children compared with iodine replete children. Thus, regardless of study design, iodine deficiency had a substantial impact on mental development. Methodological concerns included weak study designs, the omission of important confounders, small sample sizes, the lack of cluster analyses, and the lack of separate analyses of verbal and non-verbal subtests. Quantifying more precisely the contribution of iodine deficiency to delayed mental development in young children requires more well-designed randomized controlled trials, including ones on the role of iodized salt.

## 1. Introduction

Iodine is necessary for the production of thyroid hormones, thyroxine (T4) and triiodothyronine (T3) which play a major role in growth mechanisms and the development of tissues. Their action is initiated through nuclear receptors which are found in most organs [1,2]. Thyroid hormones are also involved in the regulation of the basal metabolic rate and in macronutrient metabolism [3,4]. In the central nervous system, cell migration, differentiation and myelination are also regulated by thyroid hormones [5]. Iodine deficiency disrupts the metabolism of thyroid hormones. The level of T4 decreases progressively with the severity of the deficiency in iodine following a pattern similar to serum and most tissues. However, the level of T3 (the active form of the thyroid hormones) follows different patterns of changes. In mild to moderate iodine deficiency, some adaptation mechanisms such as increased iodine trapping, increased conversion of T4 into T3, and preferential synthesis of T3 attempt to keep the concentration of T3 in the normal range. Even in severe iodine deficiency, the level of T3 is still at least normal in some tissues such as the ovary, the muscle, the lung, and the heart. The changes in other tissues like the liver and the brown adipose tissue follow a pattern similar to serum. The level of T3 in the kidney and the cerebellum decrease below the normal range only in severe iodine deficiency. The brain and the pituitary T3 level appears to be very sensitive to iodine deficiency as its level falls below the normal level even in mild or moderate iodine deficiency [6,7,8,9]. Critically low levels of thyroid hormones associated with severe iodine deficiency cause neurological damage to the brain, particularly during the fetal and neonatal period, which can result in cretinism manifested by delayed motor and mental development [10,11,12,13]. However, even mild to moderate deficiency of iodine may result in delayed mental development [14,15]. The present review examines the evidence for the effect of iodine deficiency on the mental development of children assessed at 5 years and under. This age group is particularly important given the recent research and policy attention that recognizes that 39% of children under 5 years in low-income and middle-income countries do not reach their mental potential; reasons for this include iodine deficiency [16,17]. Recent reviews of iodine-deficient children 6 to 14 years of age leave open to questions whether mental delays may start before school age, during the early childhood years.

Trials which assessed children during the school years (6 to 14 years of age) have yielded mixed findings. Several randomized controlled trials (RCT) found no advantage for school children receiving a slow-releasing capsule of iodine compared to those receiving a placebo. For example, a trial conducted in Indonesia indicated no significant difference between the treatment and placebo groups on a four-subscale test of intelligence [18]. Likewise, in Bangladesh, Huda *et al.* [19] found no differences on seven subscales but positive differences on two verbal measures. No differences on the Stanford-Binet test or on school grades were found by Bautista in Bolivia [20]. Mixed results were reported by Gordon *et al.* [15] in New Zealand and by Zimmermann *et al.* [21] in Albania, where half the subscales produced significant differences (e.g., perceptual reasoning) and half did not. Also, one study conducted in Malawi found more positive outcomes using cognitive-perceptual tasks but mixed findings for verbal ones [22]. Finally, a follow-up study of children whose mothers received or did not receive iodine supplements during pregnancy found that at 11 and 15 years of age the two groups did not differ on a perceptual reasoning task or on several fine motor tasks [23]. Inconsistent findings where half or fewer of the comparisons showed an advantage for supplemented children raise questions about which domains of mental functioning benefit from iodine supplementation in this age group, how to assess those domains, and the extent to which intervention during this period can make up for the lasting effects of iodine deficiency in utero, infancy and early childhood. It is possible that effects may be stronger if the intervention takes place earlier when the brain as well as language and cognition develop rapidly and would therefore be more affected by iodine deficiency. 

Three meta-analyses have concluded that iodine deficient populations have 13.5 [24], 8 to 10 [25] or 8.7 to 12.5 [26] intelligence quotient (IQ) points lower than iodine replete populations. The first meta-analytic review published in 1994 [24] included 18 studies with samples ranging in age from infancy to adulthood, along with a wide variety of tests including gross motor milestones, perceptual reasoning, fine motor, and full-scale intelligence tests. Only two of these studies were RCT; others simply compared villages that differed naturally in iodine sufficiency. One major limitation of observation studies comparing communities that differ in iodine sufficiency is that these communities may have differed on many other dimensions that influenced children’s mental development but were not part of the analysis. The second meta-analysis [25] included a few additional studies up to 2001 with children and adults, and gave effects sizes for intervention and observational studies separately. Eleven intervention studies with 15 effect sizes yielded a pooled effect size of *d* = 0.56 which translates into 8 IQ points, while 7 observational studies with 12 effect sizes yielded an effect size of 0.67 or 10 IQ points. The third meta-analysis [26] included 37 studies all from China with children under 16 years. Of these, six compared children from communities that differed in iodine sufficiency; the effect size of *d* = 0.83 translates to an average difference of 12.45 IQ points. These communities no doubt differed on many dimensions that might have influenced children’s mental development. The 21 studies comparing children whose mothers received iodine prenatally or children who received iodine after birth yielded an effect size of *d* = 0.58, equivalent to 8.7 IQ points. Several other reviews have added a few new studies that did not change the conclusions [27,28,29]. Given the mixed findings of studies that assessed children at 6 to 14 years of age, and the lack of similar reviews of children in the early years, we focused this review on children 5 years and under [28]. This is an important age group as cognitive and language skills are known to develop early and to be cumulative. Because a great deal of brain development occurs during the fetal stage, we also paid attention to studies that examined iodine status of pregnant mothers. 

To examine whether iodine status of mothers or infants affect mental development of young children, we reviewed studies that assessed mental development of children 5 years and under in relation to their mother’s iodine status or their own iodine status. Both intervention and cohort studies were included.

## 2. Methods

A review procedure specified in advance the study designs, the main outcome (the mental development score), the participants (children 5 years and under) as well as the data extraction. Modification of this protocol included the addition of a meta-analysis and exclusion of cross-sectional studies.

### 2.1. Study Search

An electronic literature search was conducted to identify papers on iodine and mental development outcomes in children, published from January 1980 to November 2011 on Medline. The search terms used were Bayley; child development; cognition; congenital hypothyroidism; deficiency diseases; dietary supplements; food, fortified; goiter; goiter, endemic; hypothyroidism; intelligence; iodine; iodized oil; motor skills; potassium iodide; psychomotor performance; sodium chloride, dietary; trace elements. Limiters in the database were set to (“newborn infant (birth to 1 month)” or “infant (1 to 23 months)” or “preschool child (2 to 5 years)” or “child (6 to 12 years)”). This latter age group was included in the search terms to help identify studies that include a larger age range (e.g., 0 to 12 years) with possible analysis of sub-age groups of children of 0 to 5 years. An electronic search of related citations was also performed on PubMed. The references in the identified studies were manually searched for additional studies, along with hand searches of proceedings of conferences where reports from prior to 1980 were published.

### 2.2. Inclusion and Exclusion Criteria

Inclusion criteria for this systematic review included: (1) exposure to different iodine levels before pregnancy, during pregnancy, or shortly after birth, (2) examination of iodine exposure and mental development outcome (encompassed cognitive, language and fine motor, not gross motor) of children aged 5 years and under, and (3) placebo, historical control or iodine sufficient siblings or children of similar age as a control group. Study designs included in the systematic review were: (1) randomized controlled trial with iodine supplementation of mothers; (2) non-randomized trial with iodine supplementation of mothers and/or infants; (3) prospective cohort study stratified by pregnant women’s iodine status; (4) prospective cohort study stratified by newborn iodine status. Studies on only preterm births or low/very low birth weight newborns (*k* = 4; [30,31,32,33]) were excluded because these studies were likely to produce different results due to the effect of birth weight and gestational age on the outcome of interest. Additional exclusion criteria included: (1) use of non-standardized psychometric tests (*k* = 1; [34]), (2) sole focus on gross motor milestones (*k* = 1; [35]), (3) cross-sectional observational designs that compared iodine-sufficient with iodine-deficient communities as they did not control for obvious confounds and did not provide sufficient clusters for analysis (*k* = 2; [36,37]), and (4) stratification of mothers based on an indicator of thyroid dysfunction, the thyroid peroxidase antibody (TPOAb), rather than of their iodine status (*k* = 1; [38]).

### 2.3. Study Selection and Data Extraction

Titles and/or abstracts of studies identified through the search were screened independently by two or more reviewers for eligibility against the inclusion and exclusion criteria. Data were also extracted independently from papers by two or more reviewers. Data extraction tables were created to include the following information: (1) study design; (2) sample size and age of children when mental development outcome was measured; (3) iodine biological indicators; (4) test of mental development; and (5) outcomes, group comparisons and effect size *d*. This last was the main summary measure. There was high reliability across reviewers; discrepancies were resolved through discussion. For each comparison between groups, effect size *d* and 95% confidence interval was calculated with the numerator as the difference between means and the denominator as the standard deviation (SD). SD usually ranged from 10 to 15. Where SD was not reported, the standard of 15 was used as the denominator. Different effect sizes were calculated for the same children if they were retested at later ages. The heterogeneity of study effect sizes in each design that was included in the meta-analysis was assessed by *Q* test. Pooled effect sizes were based on a random effects model when studies were heterogeneous (*Q* test; *p* < 0.05) or based on a fixed effects model when studies were homogeneous (*Q* test; *p* > 0.05). 

### 2.4. Quality Assessment of RCT

Random generation of allocation, allocation concealment, blinding, intention-to-treat analysis, attrition, success of randomization (similarity of groups at baseline), and description of outcome were included in quality assessment of RCT.

## 3. Results

### 3.1. Trial Flow

**Figure 1:**
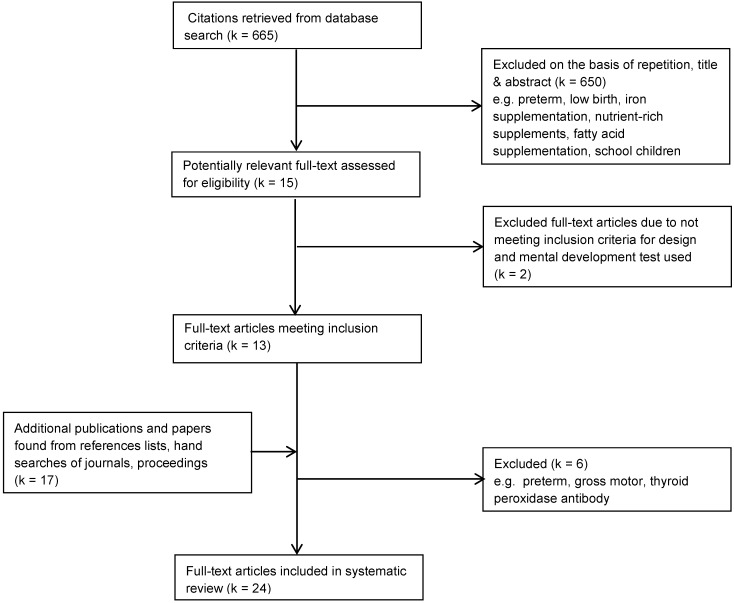
Selection of studies for systematic review of the effect of iodine on infant/child mental development.

The initial search on Medline yielded 665 citations including 15 repetitions. A review of titles and/or abstracts showed that 15 were potentially relevant. Two studies were excluded after full-text review. Additional searches identified a further 17 studies, of which 6 were excluded after full-text review. Consequently, 24 studies were included in the systematic review (Figure 1).

### 3.2. Study Characteristics

The studies included in the current review were classified into four designs: (1) RCT where individual women or communities were randomized to receive iodine supplements or a placebo; (2) non-randomized intervention trials where some women or communities were assigned to receive iodine and others received nothing; (3) prospective cohort design where women were stratified by their iodine status and followed up to delivery; (4) prospective cohort design where infants with varying iodine status due to congenital hypothyroidism (caused primarily by abnormal thyroid development (dysgenesis) and to a smaller degree by defects in thyroid hormone biosynthesis (dyshormonogenesis)) were followed up for several years. Intervention studies using the first two designs were analyzed separately as well as together because they directly compared two groups assigned to receive or not receive iodine supplements. The other two groups were examined separately. Eight of the ten intervention studies were from low-income or middle-income countries: China, Democratic Republic of Congo (DR Congo, formerly Zaire), Ecuador and Peru. Only the study from China was conducted in the 1990s; studies from the other countries were carried out in the 1960s and 1970s. Two of the ten intervention studies were conducted more recently in Spain. Of the two groups of observational designs using prospective cohorts, studies covered a longer period from the 1960s to 2011, and the majority of them were from high-income and middle-income countries and regions, such as China, North America and Europe. In the prospective cohort studies stratified based on women’s iodine status, different indicators were used to define them as euthyroid: (a) fT3 between 50th and 90th percentile, (b) TSH < 4.21 mIU/L, normal fT4 and tT4, and TPOAb- or tT4 > 101.79 nmol/L, normal TSH and fT4, and TPOAb-, (c) TSH < 4 mU/L with fT3 = 3.8 to 9.2 pmol/L and fT4 = 7.7 to 18 pmol/L, or (d) BEI = 5.5 to 10.5 µg/100 mL.

### 3.3. Mental Development Tests Used

The common measures of mental development were Bayley [39,40], Brunet-Lézine [41], and Stanford-Binet [42]. Eight of the ten intervention studies used one or more of these measures. Two others from Ecuador used the Gesell [43] which includes similar items as the other three measures. Although these are well-known, validated instruments with a sound reputation, measuring verbal and non-verbal (cognitive, fine motor) skills, most researchers reported only the total scores and not the subtest scores. Consequently, the potential separate effects of iodine on verbal, cognitive and fine motor skills were often not distinguished. Of the 16 observational studies, most used the Bayley and the McCarthy [44], along with the Brunet-Lézine, Stanford-Binet and Griffiths [45].

### 3.4. Associations between Iodine and Mental Development

Findings from the four designs are summarized in Table 1, Table 2, Table 3 where means, SD and *d* effect sizes are reported for individual studies. Studies providing supplementation to women before or during pregnancy (Table 1) tended to give injections of iodized oil of 950 mg; only the studies conducted in China and DR Congo gave lower doses (400 and 475 mg, respectively). Baseline levels of iodine status of participants, or in some cases of the whole cluster, are provided in Table 1, Table 2, Table 3. Sample sizes ranged from 12 per group to 400 (control groups were often much larger than supplemented groups, e.g., in Ramirez *et al*. [46]). The median sample size per group across the ten intervention studies was 50. Among the 10 studies where stratification was based on the mother’s iodine status (Table 2), the median sample size was 50 with a range of 7 to 624 (again groups with normal levels of iodine were often larger). Among 9 studies where stratification was based on children’s iodine status (Table 3), the median sample size was 40 with a range of 7 to 652. Three of the studies stratifying by maternal iodine status also analyzed the data by stratifying by newborn iodine status [47,48,49].

**Table 1:** Intervention studies.

Author, country	Design, treatment population and doses	Sample size	Age at testing	Biological indicator	Mental development test	Outcomes	Group comparisons (mean ± SD)	Effect size d (95% confidence interval)
*RCT*								
Pretell *et al.*, Peru [50]	Double blind RCT	*n* = 72	0–5 years	UIE, T4, T3, TSH, TI, TBG	Brunet-Lézine	0	G1 (80.0 ± 12) > Gc (75.0 ± 12)	0.41 (−0.04, 0.86)
	Iodized oil group (G1) *vs.* placebo (Gc)	G1 (*n* = 35)		Enrollment [51]	Stanford-Binet		Estimated from graph	
	W, PW in early pregnancy	Gc (*n* = 46)		UIE = 17 μg/24 h				
	Single dose of 95–950 mg Iodine			T4 = 4.1 μg/100 mL				
	Severe ID area (Goiter 83%)							
Thilly *et al.*, DR Congo [52,53,54,55]	Double blind RCT	*n* = 75	4–23 months	UIE, T4, fT4, T3, TSH, TBG	Brunet-Lézine	+	G1 (115 ± 3) > Gc (103 ± 4) **	0.99 (0.50, 1.48)
	Iodized oil group (G1) *vs.* placebo (Gc)	G1 (*n* = 39)		Enrollment				
	PW in 2st and 3nd trimester	Gc (*n* = 36)		T4 = 11.3 μg/dL				
	Single dose of 475 mg Iodine			T3 = 206 ng/dL				
	Severe ID area (Goiter 62%)			TSH = 8.2 μU/mL				
*Non-RCT*								
Berbel *et al.*, Spain [56]	Comparative post only	*n* = 44	18 months	UIE, fT4, TSH	Brunet-Lézine	+	G1 (101.8 ± 9.7) > G2 (92.2 ± 15.4) *	1.38 (0.45, 2.32)
	Treatment groups, PW?	G1 (*n* = 13)		Enrollment	Gross motor	+	G1 (101.8 ± 9.7) > Gc (87.5 ± 8.9) ***	1.39 (0.57, 2.22)
	Daily dose of 200 μg KI from enrollment (G1: 1st trimester, G2: 2nd trimester, Gc: after delivery) to end of lactation	G2 (*n* = 12)		G1 (TSH < 4.8 μU/mL; fT4 > 0.91 ng/dL)	Fine motor	+	G2 (92.2 ± 15.4) = Gc (87.5 ± 8.9)	0.31 (−0.45, 1.06)
	Mild ID area	Gc (*n* = 19)		G2 and Gc (TSH < 4.8 μU/mL; fT4 = 0.91 to 0.82 ng/dL)	Language	0		
					Socialization	+		
Cao *et al.*, China [57]	Comparative pre-post	*n* = 404	2 years?	UIE, T4, TSH?	Bayley-I	+	G1 (77 ± 11) = Gc (75 ± 18)	1.04 (0.61, 1.47)
	Iodized oil group *vs.* historical control	G1 (*n* = 28)		Enrollement			G2 (90 ± 14) > Gc( 75 ± 18) ***	0.33 (−0.06, 0.72)
	PW and Infant?	G2 (*n* = 71)		UIE = 10 to 25 μg/L			G3 (80 ± 15) = Gc (75 ± 18)	0.33 (−0.03, 0.69)
	Single dose of 400 mg iodine (PW: G1 1st trim; G2 2nd trim; G3 3rd trim); 200 mg (children > 12 months) and 50 mg (Infant: G4 0–3 months, G5 3–12 months) *vs.* historical control (Gc)	G3 (*n* = 85)					G4, G5 (80 ± 10) = Gc (75 ± 18)	
	Severe ID area (Goiter 54%)	G4 (*n* = 90)					G1 received 0.4 mg Iodine instead of 400 mg due to government new supplementation program	
		G5 (*n* = 93)						
		Gc (*n* = 37)?						
Fierro-Benitez *et al.*, Ecuador [58]	Comparative post only?	*n* = 150	41–60 months	Enrollment [59]	Stanford-Binet	+	G1 (80.1) > Gc1 (70.1) *?	0.66 (0.23, 1.09)
	Treatment cluster *vs.* control cluster (Gc)	G1 (*n* = 41)		UIE: 0.37 μg/100 mL (Treatment cluster); 0.63 μg/100 mL (Control cluster)			G2 (67.0) = Gc2 (70.1)	−0.20 (−0.73, 0.32)
	W, PW and children	Gc1(*n* = 50)					G1 (80.1) > G2 (67.0) *	0.86 (0.34, 1.39)
	Several doses: 95 mg Iodine (<2 years), 238 mg (2–6 years), 475 mg (6–12 years) and 950 mg (>12 years)	G2 (*n* = 26)						
	G1: children supplemented in early intrauterine life, in lactation and by injection; G2: children supplemented in last period (7–9th month) of fetal life, in lactation and by injection	Gc2 (*n* = 33)						
	Severe ID area (Goiter 53%–70%) [59]							
Fierro-Benitez *et al.*, Ecuador [60]	Comparative post only?	*n* = 216	3–5 years	Enrollment [59]	Stanford-Binet	+	G1 (83.66 ± 13.4) > Gc1 (72.74 ± 14.0) **?	0.81 (0.45, 1.19)
	Treatment cluster *vs.* control cluster (Gc)	G1 (*n* = 63)		UIE: 0.37 μg/100 mL (Treatment cluster); 0.63 μg/100 mL (Control cluster)			G2 (71.72 ± 14.6) = Gc2 (69.16 ± 13.3)	0.18 (−0.24, 0.60)
	W and PW	Gc1(*n* = 63)					G1 (83.66 ± 13.4) *vs.* G2 (71.72 ± 14.6)	0.79 (0.37, 1.21)
	Several doses: 95 mg Iodine (<2 years), 238 mg (2–6 years), 475 mg (6–12 years) and 950 mg (>12 years)	G2 (*n* = 40)						
	G1: Supplementation prior to conception, in lactation and by injection; G2: Supplementation in 4–7th month of fetal life, in lactation and by injection	Gc2 (*n* = 50)						
	Severe ID area (Goiter 53%–70%) [59]							
Ramirez *et al.*, Ecuador [61]	Comparative post only	*n* = 227	9, 13 and 18 months	UIE, T4, TI, PBI, BEI, BII [59]	Gesell	0	G1 (92.77) = Gc (89) [62]	0.25 (−0.03, 0.53)
	Treatment cluster (G1) *vs.* control cluster (Gc)	G1 (*n* = 72)		Enrollment [59]				
	PW	Gc (*n* = 155)		UIE: 0.37 μg/100 mL (Treatment cluster); 0.63 μg/100 mL (Control cluster)				
	Single dose of 950 mg Iodine [59]							
	Severe ID area (Goiter 53%–70%) [59]							
Ramirez *et al.*, Ecuador [46]	Comparative post only	*n* = 583	3–60 months	Enrollment [59]	Gesell	0	G1 (89.7) *vs.* Gc (87.4) (Estimated)	0.15 (−0.02, 0.33)
	Treatment cluster (G1) *vs.* control cluster (Gc)	G1 (*n* = 183)		UIE: 0.37 μg/100 mL (Treatment cluster); 0.63 μg/100 mL (Control cluster)				
	W and PW (0th–5th month)	Gc (*n* = 400)						
	Single dose of 950 mg Iodine							
	Severe ID area (Goiter 53%–70%) [59]							
Trowbridge, Ecuador [63]	Comparative post only	*n* = 125	3–5 years	Enrollment [59]	Stanford-Binet	0	G1 (76.8) = Gc1 (72.4)	0.29 (−0.31, 0.89)
	Treatment cluster *vs.* control cluster (Gc)	G1 (*n* = 22)		UIE: 0.37 μg/100 mL (Treatment cluster); 0.63 μg/100 mL (Control cluster)			G2 (72.3) = Gc2 (69.0)	0.22 (−0.40, 0.83)
	W, PW, and Infant?	Gc1 (*n* = 24)					G3 (65.2) = Gc3 (69.9)	−0.31 (−1.00, 0.39)
	Single dose of 950 mg Iodine [64] (G1 prior to conception; G2 during pregnancy), 95 mg Iodine (G3 between 0 and 9 months of age)	G2 (*n* = 21)					G1 (76.8) > G3 (65.2) **	
	Severe ID area (Goiter 53%–70%) [59]	Gc2 (*n* = 23)						
		G3 (*n* = 16)						
		Gc3 (*n* = 19)						
Velasco *et al.*, Spain [65]	Comparative post only	*n* = 194	3–18 months	UIE, fT4, fT3, TSH, Tg	Bayley-I	0	G1 (109.22 ± 11.73) = Gc (108.9 ± 13.41)	0.02 (−0.28, 0.33)
	Treatment group (G1) *vs.* control group (Gc)	G1 (*n* = 133)		Enrollment				
	PW, postpartum W	Gc (*n* = 61)		G1: 153–213 μg/L (UIE); 8.8–10.6 pmol/L (fT4)				
	Daily dose of 300 μg iodine (KI) from 1st trimester through lactation			Gc: 87.6 (UIE); 9.0 pmol/L (fT4)				
	Moderate ID area							

Notes: RCT (Randomized controlled trial); Effect size *d* (Standardized mean difference, SMD); SD (standard deviation); ID (iodine deficiency); KI (Potassium iodide); PW (Pregnant women); W (women of child bearing age); G1, G2, …, Gc (Group 1, Group 2, …, Group control); UIE (Urinary iodine excretion); T4 or tT4 (Thyroxine); fT4 (free thyroxine); T3 or tT3 (triiodothyronine); tT3 (free triiodothyronine); TSH (Thyroid stimulating hormone); Tg (Thyroglobuline); TBG (Thyroxine binding globulin); BII (Butanol insoluble iodine); TI (serum iodine); PBI (Protein-bound iodine); BEI (Butanol extractable iodine); Outcome: + significant group difference, 0 no significant group difference; * *p* < 0.05; ** *p* < 0.01; *** *p* < 0.001.

**Table 2:** Cohort prospective studies stratified by maternal iodine status.

Author, country	Design, groups	Sample size	Age at testing	Biological indicator	Mental development test	Outcomes	Group comparisons (mean ± SD)	Effect size d (95% confidence interval)
Costeira *et al.*, Portugal [66]	Cohort Prospective	*n* = 86	12, 18 and 24 months	UIE, tT4, fT4, tT3, fT3, TSH, at 1st, 2nd and 3rd trimester	Bayley-I 12 months	+	G1 (77.7 ± 17.9) < Gc (99.3 ± 17.6) *	1.18 (0.37, 1.99)
	G1 (hypothyroid: fT3 < 10th percentile) at 3rd trimester	G1 (*n* = 9)			Bayley-I 24 months	0	G1 (91.1 ± 21.4) = Gc (100.7 ± 17.2)	0.52 (−0.25, 1.29)
	Gc (euthyroid: fT3 between 50th and 90th percentile) at 3rd trimester	Gc (*n* = 33)						
	Moderate ID area							
Galan *et al.*, Spain [47]	Cohort Prospective	*n* = 61	37–47 months (Mea*n* = 40)	UIE, fT4, TSH, TPOAb at 1st and 3rd trimester	McCarthy	+	G1 (97.7) < Gc (105.5) *	0.64 (0.12, 1.17)
	G1 (UIE < 200 μg/L at 12 weeks)	G1 (*n* = 30)			Verbal	+	G1 (49.4) < Gc (55.2) *	
	Gc (UIE ≥ 200 μg/L at 12 weeks)	Gc (*n* = 31)			Perceptual	0	G1 (50.1) = Gc (54.1)	
	Moderate ID area				Quantitative	0	G1 (45.7) = Gc (45.9)	
					Memory	0	G1 (46.0) = Gc (49.4)	
					Motor	0	G1 (52.8) = Gc (55.6)	
	G1 (non-Iodized salt at 12 weeks)?	G1 (*n* = 24)			McCarthy	+	G1 (97.59) < Gc (105.36) *	
	Gc (Iodized salt at 12 weeks)	Gc (*n* = 37)			Verbal	+	G1 (49.76) < Gc (54.96 ) *	
	Moderate ID area				Perceptual	+	G1 (48.88) < Gc (53.84) *	
					Quantitative	0	G1 (45.94) = Gc (47.04)	
					Memory	0	G1 (46.18) = Gc (49.80)	
					Motor	+	G1 (49.06) < Gc (56.72) *	
Li *et al.*, China [67]	Cohort Prospective	*n* = 111	25–30 months	tT4, fT4, TSH, TPOAb at 16–20 weeks	Bayley-I	+	G1, G2 (111.1) < Gc (120.2)	0.75 (0.34, 1.17)
	G1 & G2 (hypothyroid)	G1 (*n* = 18)						
	Gc (euthyroid: TSH < 4.21 mIU/L, normal fT4 and tT4, TPOAb− or tT4 > 101.79 nmol/L, normal TSH and fT4, TPOAb−)	G2 (*n* = 19)						
	Iodine sufficiency area	Gc (*n* = 74)						
Man *et al.*, USA [68,69]	Cohort Prospective	*n* = 326	8 months	BEI at 12–29 weeks	Bayley-I	+	G1 (95) *vs.* Gc (101)	0.40 (0.10, 0.70)
	Hypothyroxynemic mothers inadequately treated (G1), adequately treated (G2)	G1 (*n* = 55)					G1 (95) < G2 (102) **	
	Gc (euthyroid mothers: BEI = 5.5 to 10.5 μg/100 mL)	G2 (*n* = 29)						
	Iodine sufficiency area	Gc (*n* = 242)						
		G1 (*n* = 23)	4 years		Stanford-Binet	+	G1 (93 ± 15.9) *vs.* Gc (100)	0.47 (0.03, 0.90)
		G2 (*n* = 22)					G1 (93 ± 15.9) < G2 (102 ± 14.7) *	
		Gc (*n* = 227)						
Murcia *et al.*, Spain [48]	Cohort Prospective	*n* = 674?	11–16 months	Iodine intake, IS at 1st and 3rd trimester	Bayley-I	0	G1 (100.13 ± 15) = Gc (99.6 ± 16.5)	−0.02 (−0.18, 0.15)
	Iodine intake from multivitamin supplement (Iodine)	G1(*n* = 467)		UIE, fT4, TSH, in 1st trimester				
	G1 (Iodine < 150 μg/day)	Gc(*n* = 222)						
	Gc (Iodine ≥ 150 μg/day)?							
	G1 (UIE < 150 μg/L)	G1 (*n* = 357)				0	G1 (100.38 ± 15)= Gc (99.10 ± 15)	−0.09 (−0.24, 0.07)
	Gc (UIE > 150 μg/L)	Gc (*n* = 292)						
	G1 (non-Iodized salt)	G1 (*n* = 251)				0	G1 (100.3 ± 15.0) = Gc1 (99.8 ± 15.0)	−0.03 (−0.19, 0.12)
	Gc1 (Iodized salt)	Gc1 (*n* = 440)						
	G1 (TSH > 4 μU/mL)	G1(*n* = 24)				0	G1 (104.0 ± 13.5) = Gc (100.0 ± 14.8)	−0.27 (−0.70, 0.17)
	Gc (TSH ≤ 4 μU/mL)	Gc (*n* = 624)						
	Iodine sufficient and mild deficient areas							
Oken *et al.*, USA [49]	Cohort Prospective	*n* = 500	6 months?	T4, TSH, TPOAb	Visual recognition memory (VRM)	0	62.9 ± 16.0	0.10 estimated
	Correlations with Mother’s history of thyroid disease							
	Iodine sufficient area		3 years		Peabody picture vocabulary test (PPVT)	+	106 ± 13.2	
Pop *et al.*, Netherlands [70]	Cohort Prospective	*n* = 220		fT4, TSH, TPOAb at 12 weeks of gestation	Bayley-I	0	G1 (110) = Gc (115)	0.33 (−0.11, 0.78)
	G1 (fT4 ≤ 10th percentile)	G1 (*n* = 22)					Estimated from graphs	
	Gc (fT4 > 10th percentile)	Gc (*n* = 198)						
	Iodine sufficient area							
Pop *et al.*, Netherlands [71]	G1 (fT4 ≤ 10th percentile)	G1 (*n* = 57)	2 years		Bayley-I	+	G1 (98 ± 15) < Gc (106 ± 14) *	0.53 (0.15, 0.91)
	Gc (50th ≤ maternal fT4 ≤ 90th)	Gc (*n* = 58)						
	Iodine sufficient area							
	G1 (fT4 ≤ 10th percentile)	G1 (*n* = 57)	2 years		Bayley-I	+	G1 (98 ± 15) < Gc (106 ± 14) *	0.53 (0.15, 0.91)
	Gc (50th ≤ maternal fT4 ≤ 90th)	Gc (*n* = 58)						
	Iodine sufficient area							
Radetti *et al.*, Italy [72]	Cohort Prospective	*n* = 29	9 months	T4, fT4, T3, fT3, TSH, TBG, Tg, TPOAb?	Brunet-Lézine	0	G1 (99 ± 6.5) = Gc (98 ± 6.4)	−0.08 (0.91, 0.74)
	G1 (treated for hypothyroidism)	G1 (*n* = 9)			Motor	0		
	Gc (euthyroid: TSH < 4 mU/L with fT3 = 3.8 to 9.2 pmol/L and fT4 = 7.7 to 18 pmol/L)	Gc (*n* = 20)			Social dev.	0		
	ID area				Speech	0		
					Eye-Hand coord	0		
Smit *et al.*, Netherlands [73]	Cohort Prospective	*n* = 20	6 months	T4, T3 and TSH at 1st, 2nd and 3rd trimester	Bayley-I	+	G1 (95.7 ± 14.7) < Gc (112.4 ± 13.2) *	1.04 (−0.30 ,2.37)
	G1 (hypo or subclinical hypothyroid)	G1 (*n* = 7)						
	G2 (hyper or subclinical hyperthyroid)	G2 (*n* = 7)						
	Gc (euthyroid: 1st trim: TSH = 0.3 to 2.0 mU/L; fT4 = 7.4 to 24.2 pmol/L; T3 = 2.0 to 3.6 nmol/L; 2nd trim: TSH = 0.5 to 2.3 mU/L; fT4 = 5.1 to 14.3 pmol/L; T3 = 2.2 to 3.8 nmol/L)	Gc (*n* = 6)	12 months		Bayley-I	+	G1 (107.8 ± 15.3) < Gc (123.5 ± 17.8) *	0.97 (−0.35, 2.30)
	Iodine sufficient area		24 months		Bayley-I	0	G1 (104.2 ± 31.0) = Gc (110.6 ± 29.6)	0.40 (−0.85, 1.64)

Notes: RCT (Randomized controlled trial); Effect size *d* (Standardized mean difference, SMD); SD (standard deviation); ID (iodine deficiency); KI (Potassium iodide); PW (Pregnant women); W (women of child bearing age); G1, G2, …, Gc (Group 1, Group 2, …, Group control); UIE (Urinary iodine excretion); T4 or tT4 (Thyroxine); fT4 (free thyroxine); T3 or tT3 (triiodothyronine); tT3 (free triiodothyronine); TSH (Thyroid stimulating hormone); TPOAb (Thyroid peroxidase antibody); Tg (Thyroglobuline); TBG (Thyroxine binding globulin); BII (Butanol insoluble iodine); TI (serum iodine); PBI (Protein-bound iodine); BEI (Butanol extractable iodine); Outcome: + significant group difference, 0 no significant group difference; * *p* < 0.05; ** *p* < 0.01; *** *p* < 0.001.

**Table 3:** Cohort prospective studies stratified by infant iodine status.

Author, country	Design, groups	Sample size	Age at testing	Biological indicator	Mental development test	Outcomes	Group comparisons (mean ± SD)	Effect size d (95% confidence interval)
Bongers-Schokking *et al.*, Netherlands [74]	Cohort Prospective	*n* = 61	10–30 months	Newborn with CH (TSH, fT4, TBG,Tg) treated with thyroxine	Bayley-I	+	G1, G2, G3, G4 (106 ± 19) < G5, G6, G7, G8 (118 ± 11) ***	0.80 (0.25, 1.33)
	G1(severe CH early treat, high dose)	G1 (*n* = 7)				+	G1, G2 (112.19 ± 13) > G3, G4 (98.09 ± 21)	0.91 (0.06, 1.76)
	G2 (severe CH early treat, low dose)	G2 (*n* = 9)						
	G3 (severe CH late treat, high dose)?	G3 (*n* = 6)						
	G4 (severe CH, late treat, low dose)	G4 (*n* = 5)						
	G5 (mild CH early treat, high dose)	G5 (*n* = 5)						
	G6 (mild CH early treat, low dose)	G6 (*n* = 7)						
	G7 (mild CH late treat, high dose)	G7 (*n* = 11)						
	G8 (mild CH, late treat, low dose)	G8 (*n* = 11)						
Choudhury and Gordon, China [75]	Cohort Prospective	*n* = 275	7 months	Cord TSH	Fagan TII	0	G1 (58.9 ± 4.3) = Gc (59.6 ± 3.0)	0.19
	G1 (TSH = 10.0–29.9 mU/L)	G1 (*n* = 94)				+	G2 (57.7 ± 5.6) < Gc (59.6 ± 3.0)	
	G2 (TSH = 20.0–29.9 mU/L)	G2 (*n* = 82)				+	G3 (57.5 ± 3.1) < Gc (59.6 ± 3.0)	0.66 (0.25, 1.08)
	G3 (TSH ≥ 30.0 mU/L)	G3 (*n* = 41)						
	Gc (controls: TSH < 5 mU/L)	Gc (*n* = 58)						
	Mild ID area	*n* = 135	13 months		Bayley-II	+	G1 (98.2 ± 8.3) < Gc (102.5 ± 8.2) *	0.74 (0.14, 1.34)
						+	G2 (98.7 ± 9.3) < Gc (102.5 ± 8.2) *	
						+	G3 (93.5 ± 11.1) < Gc (102.5 ± 8.2) *	
Galan *et al.*, Spain [47]	Cohort prospective	*n* = 61	37–47 months (Mea*n* = 40)	Neonatal TSH	McCarthy	+	G1 (95.1 ± 12) < Gc (104.9) *	0.81 (0.14, 1.5)
	G1 (TSH ≥ 5 mU/L)	G1 (*n* = 12)			Verbal	+	G1 (49.2 ± 7.4) < Gc (56.9) *	1.04
	Gc (TSH < 5 mU/L)	Gc (*n* = 49)			Perceptual	+	G1 (48.6 ± 8.7) < Gc (54.2) *	0.64
	Moderate ID area				Quantitative	0	G1 (43.7 ± 7.2) = Gc (47.3)	0.5
					Memory	+	G1 (43.3 ± 8.1) < Gc (50.1) *	0.84
					Motor	0	G1 (50.9 ± 9.9) = Gc (55.1)	0.42
Murcia *et al.*, Spain [48]	Cohort Prospective	*n* = 680	11–16 months	Neonate TSH	Bayley-I	0	G1 (96.2 ± 17.0) = Gc (100.2 ± 14.9)	0.27 (™0.11, 0.65)
	G1 (TSH > 4 μU/mL)	G1 (*n* = 28)						
	Gc (TSH ≤ 4 μU/mL)	Gc (*n* = 652)						
	Iodine sufficient and mild deficient areas							
Oken *et al.*, USA [49]	Cohort Prospective	*n* = 500	6 months?	Newborn T4	Visual recognition memory (VRM)	0	62.9 ± 16.0	0.10 estimated
	Used newborn T4 as continuous variable							
	Iodine sufficient area		3 years		Peabody picture vocabulary test	0	106.0 ± 13.2	0.10 estimated
					Fine Motor			
						0	99.8 ± 11.8	0.00 estimated
Rovet *et al.*, Canada [76]	Cohort Prospective	*n* = 80	12 months	All Newborn with CH -TSH; Bone age used to determine Iodine sufficiency as a fetus	Griffiths	0	G1 (110.5 ± 10.3) = Gc (113.3 ± 10.4)	0.23 (™0.22, 0.68)
	G1 (delayed skeletal maturity (fetal hypothyroidism)	G1 (*n* = 45)			Reynell language	0	No group means given	0
	Gc (non-delayed skeletal maturity, i.e., likely iodine sufficient as a fetus)	Gc (*n* = 35)						
	All treated at birth	G1 (*n* = 31)	2 years		Griffiths	+	G1 (109.0 ± 11.2) < Gc (118.9 ± 11.8) *	0.81 (0.19, 1.43)
		Gc (*n* = 18)			Reynell language	0	No group means given	0
		G1 (*n* = 28)	3 years		Griffiths	+	G1 (111.9 ± 13.1) < Gc (121.0 ± 10.5) *	0.75 (0.14, 1.36)
		Gc (*n* = 20)			Reynell language	0	No group means given	0
					Beery-Buktenica fine motor	+	G1 (58.0 ± 5.1) < Gc (76.4 ± 4.0) *	4
					McCarthy percept	+	G (53.4 ± 2.2) < Gc (59.6 ± 2.0) *	2.95
		G1 (*n* = 20)	4 years		McCarthy	+	G1 (103.1 ± 14.7) < Gc (114.6 ± 11.1) *	0.80 (0.11, 1.5)
		Gc (*n* = 17)			Reynell RecLang	+	G1 (™0.058 ± 1.2) < Gc (0.868 ± 0.8) **	0.93
					Reynell ExpLang	+	G1 (™0.032 ± 1.0) < Gc (0.713 ± 1.0) *	1.5
					Beery-Buktenica fine motor	0	No group means given	0
		G1 (*n* = 18)	5 years		WPPSI-I	+	G1 (97.8±15) < Gc (109.2±13.1) *	0.74 (0.02,1.47)
		Gc (*n* = 16)			Reynell RecLang	0	No group means given	0
					Reynell ExpLang	+	G1 (-0.233±1.2) < Gc (0.692±0.5) *	0.93
					Beery-Buktenica fine motor	+	G1 (42.3±5.6) < Gc (62.4±6.2) *	3.35
Rovet *et al.*, Canada [77], Study 1	Cohort Prospective	G1 (*n* = 108)	12–18 months	Newborn with CH (TSH)	Griffiths	0	G1 (111.0) = Gc (109.4)	™0.11 (™0.41, 0.20)
	G1(CH treated with 8–10 μg/kg l-thyroxine)	Gc (*n* = 71)			Bayley-I	+	G1 (106.4) < Gc (113.3)	0.46 (0.15, 0.76)
	Gc (Control siblings)		2 years		Griffiths	0	G1 (112.8) = Gc (114.1)	0.09 (™0.22, 0.39)
			3 years		Griffiths	0	G1 (114.8) = Gc (115.6)	0.05 (™0.25, 0.36)
					Reynell Language		No group means given	
			4 years		McCarthy	0	G1 (109.3) = Gc (114.0)	0.31 (0.01, 0.62)
					Reynell Language		No group means given	
			5 years		WPPSI	+	G1 (105.7) < Gc (114.5) *	0.58 (0.28, 0.89)
					Reynell language			
Rovet *et al.*, Canada [77] Study 2	Cohort Prospective	*n* = 108	12 months	Newborn with CH (TSH)	Griffiths	0	G1 (110.9 ± 15) = Gc (112.7)	0.12
	G1 (delayed skeletal maturity (fetal hypothyroidism))			Bone age				
	Gc (non-delayed skeletal maturity)		18 months		Bayley-I	+	G1 (102.5 ± 15) < Gc (116.3) *	0.92
			2 years		Griffiths	+	G1 (110.8 ± 15) < Gc (117.3) *	0.43
			3 years		Griffiths	+	G1 (112.1 ± 15) < Gc (119.6) **	0.5
			4 years		McCarthy	+	G1 (107.3 ± 15) < Gc (114.7) *	0.49
			5 years		WPPSI-I	+	G1 (104.0 ± 15) < Gc (109.8) *	0.39
Tillotson *et al.*, UK [78]	Cohort Prospective	*n* = 676	5 years	Newborn with CH treated *vs.* control	WPPSI		G1 (106.4 ± 15) = Gc (113.2)	0.45 (0.30, 0.61)
	G1 (Congenital hypothyroid, treated)	G1 (*n* = 361)						
	Gc (Control)	Gc (*n* = 315)						

Notes: RCT (Randomized controlled trial); Effect size *d* (Standardized mean difference, SMD); SD (standard deviation); ID (iodine deficiency): iodine status of area/population in studies of Bongers-Schokking *et al*. [74], Rovet *et al*. [76,77], and Tillotson *et al*. [78], not provided; KI (Potassium iodide); PW (Pregnant women); W (women of child bearing age); CH (Congenital hypothyroidism); G1, G2, …, Gc (Group 1, Group 2, …, Group control); UIE (Urinary iodine excretion); T4 or tT4 (Thyroxine); fT4 (free thyroxine); T3 or tT3 (triiodothyronine); tT3 (free triiodothyronine); TSH (Thyroid stimulating hormone); TPOAb (Thyroid peroxidase antibody); Tg (Thyroglobuline); TBG (Thyroxine binding globulin); BII (Butanol insoluble iodine); TI (serum iodine); PBI (Protein-bound iodine); BEI (Butanol extractable iodine); Outcome: + significant group difference, 0 no significant group difference; * *p* < 0.05; ** *p* < 0.01; *** *p* < 0.001.

#### 3.4.1. Intervention RCT

In a double blind RCT in Peru, Pretell *et al*. [50] found that children from women supplemented prior to conception with a single dose of 95 to 950 mg of iodized oil did not differ in their scores on the Brunet-Lézine and Stanford-Binet from children of the control group whose mothers received a placebo (Estimated mean 80.0 *vs.* 75.0; *ns*, *d* = 0.41). In the double blind RCT from the DR Congo, Thilly *et al*. [52,53,54,55] found that supplementation during the second and third trimester with a single dose of 475 mg of iodine resulted in a statistically significant difference in children’s mental outcome compared to the placebo control group (Mean 115 *vs.* 103; *p* < 0.01, *d* = 0.99). The average effect size from these two double blind RCT was *d* = 0.68.

The reporting of study details by Pretell *et al*. [50] in Peru suggests that participants were randomized but there is no detail on blinding or concealment of allocation. There also appeared to be a post-hoc selection of children whose mothers received iodine prior to conception. In the study by Thilly *et al*. [52,53,54,55], pregnant women were randomly allocated to condition and both participants and researchers were blind to treatment. The analysis excluded children between 24 and 36 months of age and the researchers reported a 40% lost-to-follow-up. Both of the analyses reported here appear to be based on intention-to-treat. 

#### 3.4.2. Intervention Non-RCT

Results from eight intervention studies where groups and individuals were not randomly assigned yielded mixed results. Overall differences were greater when supplementation occurred prior to gestation or during the first two trimesters of pregnancy; results are reported by timing of intervention.

*Supplementation Prior to or Early in Pregnancy* *vs.* *Control.* Seven out of the eight non-randomized group studies compared children of mothers supplemented prior to or early in pregnancy with a control group who did not receive iodine during pregnancy. Studies with positive outcomes are described first. For example, a non-randomized study in Ecuador by Fierro-Benitez *et al*. [60] indicated that children of women supplemented prior to gestation had a greater mean Stanford-Binet score than children of a control group (Mean 83.7 *vs.* 72.7; *p* < 0.002, *d* = 0.82). They found similar results when pregnant women were supplemented in early pregnancy (Mean 80.1 *vs.* 70.1; *p* < 0.05, *d* = 0.66) [58]. In China, Cao *et al*. [57] found that children whose mothers were supplemented with a single dose of 400 mg of iodine had a higher score than children of the same age in the same community assessed before the supplementation (historical baseline) (Mean 90 *vs.* 75; *p* < 0.001, *d* = 1.04). Berbel and coworkers [56] had an early supplemented group *vs.* one supplemented only during lactation (here considered as the comparison group). The children from the group supplemented early scored significantly higher than the comparison (Mean 101.8 *vs.* 87.5; *p* < 0.0001, *d* = 1.39). However, Ramirez *et al*. [61] found no significant difference on mental development outcomes between children of women supplemented before or during pregnancy with a single dose of 950 mg of iodine and children in the control village (Mean 92.8 *vs.* 89.0; *ns*, *d* = 0.25). Likewise no significant difference was found in a similar study where children of pregnant women supplemented before the fifth month of gestation were compared with those in the control village (estimated Mean 89.7 *vs.* 87.4; *ns*, *d* = 0.15) [46]. Trowbridge also did not find any significant difference between groups when he compared children of women supplemented prior to conception with a single dose of 950 mg of iodine with children from the control group (Mean 76.8 *vs.* 72.4; *ns*, *d* = 0.29) [63]. Velasco *et al*. [65] reported no difference in mental development scores between children of pregnant women supplemented with a daily dose of 300 µg iodine from first trimester through lactation compared with children of non-supplemented pregnant mothers (Mean 109.2 *vs.* 108.9; *ns*, *d* = 0.02). 

The average effect size across these eight non-RCT that compared iodine supplementation prior to or early in pregnancy *vs.* control was *d* = 0.51. Some had very high effect sizes but four studies showed no differences.

*Supplementation Late in Pregnancy* *vs.* *Control.* There was a consistent finding of no effect across studies that provided supplementation during late pregnancy. Fierro-Benitez *et al*. [58] reported that mental development was not different between children whose mothers were supplemented in the last trimester and their age-matched control group (Mean 67.0 *vs.* 70.1; *ns*, *d* = −0.20). Mental development was also found to be similar between children of women supplemented with 950 mg of iodine during the second trimester of pregnancy and children of women not supplemented (Mean 71.7 *vs.* 69.2; *ns*, *d* = 0.18) [60]. Likewise, no significant difference was found in China between children of mothers supplemented in the third trimester and children of the control group (Mean 80 *vs.* 75; *ns*, *d* = 0.33) [57]. Berbel *et al*. [56] reported no difference in mental development scores between children of pregnant women supplemented in the second trimester and children from the control group (Mean 92.2 *vs.* 87.5; *ns*, *d* = 0.31).

The average effect size across these four non-RCT that compared iodine supplementation late in pregnancy *vs.* control was *d* = 0.17. Effect sizes ranged from −0.20 to 0.33, with non-significant differences between groups.

*Timing of Supplementation.* Studies that compared mental development of children from women supplemented prior to gestation or early in pregnancy *vs*. late in pregnancy differ from the two previous comparisons in that generally all the participating women whose children’s mental development is compared here came from the same communities. They received supplements early or late in pregnancy due to the timing of supplements arriving in the community or the timing of their visit to the clinic, not due to the design of the study. Findings from two studies showed a fairly consistent benefit for early supplementation. Fierro-Benitez *et al*. [58] found that children whose mothers were supplemented in early pregnancy scored higher than children supplemented in the last trimester of fetal life, during lactation and directly as a newborn (Mean 80.1 *vs.* 67.0; *p* < 0.05, *d* = 0.86). In another study [60] they reported mental development means of children whose mothers were supplemented before conception and those supplemented in the second trimester (Mean 83.7 *vs.* 71.7; *p* not reported, *d* = 0.79). 

The average effect size across these two non-RCT that compared iodine supplementation early in pregnancy *vs*. late in pregnancy was *d* = 0.82.

**Figure 2:**
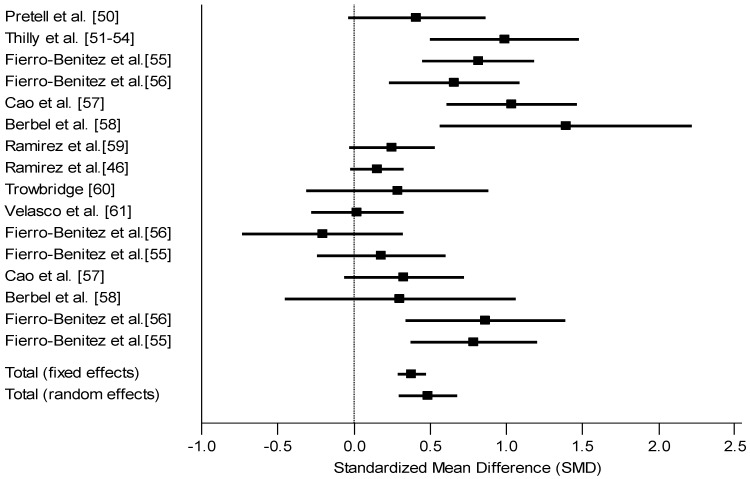
Forest plot for effect size (Standard mean difference SMD and 95% confidence interval) of iodine on mental development of children, intervention studies (The studies were heterogeneous (*Q* = 54.81, *df* = 15, *p* < 0.0001). The random effects model was therefore more appropriate).

*Summary of Intervention Studies.* Intervention studies on the effects of iodine supplements on mental outcome of children 5 years old and under showed relatively consistent findings regardless of the regional or population level of iodine deficiency (Figure 2). The average effect size for randomized (*d* = 0.68) and non-randomized (*d* = 0.46) designs was 0.49. The IQ point difference was calculated assuming a standard deviation of 15 for most intelligence tests. Consequently, an effect size of 0.49 translates into a 7.4 IQ point difference (0.49 × 15) between groups. This included a total of 16 effects across 10 studies. Most of them (15 out of 16) indicated a positive effect size. Most had targeted different periods for the supplementation, from the period prior to conception to lactation, the majority offered supplementation some time during pregnancy. Supplementation prior to or during early pregnancy benefited children more than supplements late in pregnancy. Two studies used a random assignment design and only one found a significant effect of iodine supplementation.

#### 3.4.3. Observational Cohort Prospective Studies Stratified by Maternal Iodine Status

**Figure 3:**
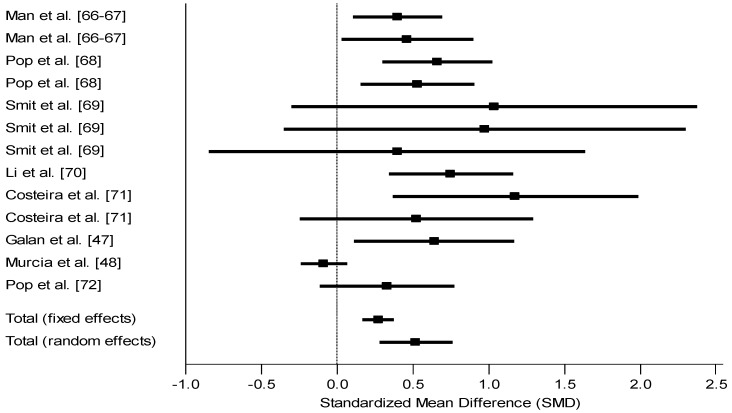
Forest plot for effect size (Standard mean difference SMD and 95% confidence interval) of iodine on mental development of children, cohort prospective studies stratified by maternal iodine status. The studies are heterogeneous (*Q* = 34.85, *df* = 12, *p* < 0.001). The random effects model was therefore more appropriate. Estimated effect size of 0.1 in Oken’s study not included as details for computation not reported.

Mothers with normal levels of iodine in the first trimester, as assessed by various iodine biological indicators, had children with consistently better mental development outcomes. Ten studies are reported on here. Man [68,69] reported that scores of children of hypothyroxinemic inadequately treated pregnant women were different from scores of children of euthyroid pregnant women (Mean 95 *vs.* 101; *p* not reported, *d* = 0.40) and similarly differed from those born to adequately treated mothers (Mean 95 *vs.* 102; *p* < 0.01, *d* = 0.47). Maternal iodine status was measured here by serum butanol-extractable iodine during pregnancy. Pop *et al*. [71] found that at 1 year of age, children of mothers with free thyroxine (fT4) between the 50th and 90th percentile at 12 weeks of gestation had a higher score than children of mothers with fT4 below the 10th percentile (Mean 105 *vs.* 95; *p* < 0.01, *d* = 0.66). The difference among the same children was also significant at 24 months of age (Mean 106 *vs.* 98; *p* < 0.05, *d* = 0.53). Smit *et al*. [73] reported that euthyroid mothers during the first two trimesters had children with higher Bayley mental scores (Mean 112.4 *vs.* 95.7; *p* < 0.05, *d* = 1.04 at 6 months; Mean 123.5 *vs.* 107.8; *p* < 0.05, *d* = 0.97 at 12 months). Li *et al*. [67] in China found a difference between children from hypothyroid and euthyroid mothers between 25 and 30 months (Mean 111.1 *vs.* 120.2; *p* not reported, *d* = 0.75). Costeira *et al*. [66] in Portugal found differences in children of 12 months but not at 24 months as a function of their mother’s level of fT3 during pregnancy (Mean 77.7 *vs.* 99.3; *p* < 0.05, *d* = 1.2 for 12 months; Mean 91.1 *vs.* 100.7; *ns*, *d* = 0.52 for 24 months). Although other comparisons were made based on other maternal indicators at different trimesters, approximately half of the comparisons were significantly different. Riano Galan *et al*. [47] found a difference on the overall McCarthy scales for children of mothers with urinary iodine excretion (UIE) above and below the cut-off (Mean 105.5 *vs.* 97.2; *p* < 0.05, *d* = 0.64); this difference was reflected in verbal but not non-verbal scores. Similarly when these same children were stratified base on their mothers’ consumption or not of iodized salt, children of mothers who used iodized salt had higher McCarthy scores; given that these are the same children simply grouped in an overlapping manner, their effect size will not be counted twice in the final tally. However, Murcia *et al*. [48] reported that children of iodine-sufficient and deficient mothers in the first trimester of pregnancy, based on UIE, had similar mental development scores (Mean 100.38 *vs.* 99.10; *p* not reported, *d* = −0.09). Oken *et al*. [49] also found that scores did not differ between children of mothers with T4 in the highest decile and children of mothers with T4 in the lowest decile in the first trimester of pregnancy (the report provided only one mean for the entire sample, 62.9; So we estimated *d* at 0.10). Similarly, Pop *et al*. [70] found no differences on the mental Bayley test between children whose mothers were deficient at 12 weeks of pregnancy, using ≤10th percentile for fT4, compared to those whose mothers were greater than the cut-off (estimated Mean 110 *vs.* 115; *p* not reported, *d* = 0.33). A study by Radetti *et al*. [72] in Italy treated iodine-deficient mothers during pregnancy and found that their children did not differ from euthyroid mothers at 9 months of age (*d* = −0.08). This study will not be included in our summary of the section. Unlike other studies in this section, mothers here were treated and their children compared with a normal group; consequently to test the efficacy of the treatment, the authors expected and found no difference between the children of treated mothers and children of normal mothers. Thus, the overall effect size from these nine studies with 14 comparisons was 0.52, with a range of −0.09 to 1.2 (Figure 3). The difference between groups was therefore 7.8 IQ points.

#### 3.4.4. Observational Cohort Prospective Studies Stratified by Newborn Iodine Status

These studies include children with congenital hypothyroidism where the thyroid gland is not functioning at birth, generally defined in terms of high levels of thyroid stimulating hormone (TSH). This is normally detected during screening at birth in high-income regions. The maternal iodine status of the children was not reported in these studies. In high-income countries, children are usually treated immediately upon detection (Table 3). Control or comparison groups of children were iodine-sufficient siblings or mildly deficient children. Thus, the hypothesis of the researchers of these studies was that infants treated with the thyroid hormone after screening and confirmation of congenital hypothyroidism at birth would have a development similar to that of infants without congenital hypothyroidism. 

**Figure 4:**
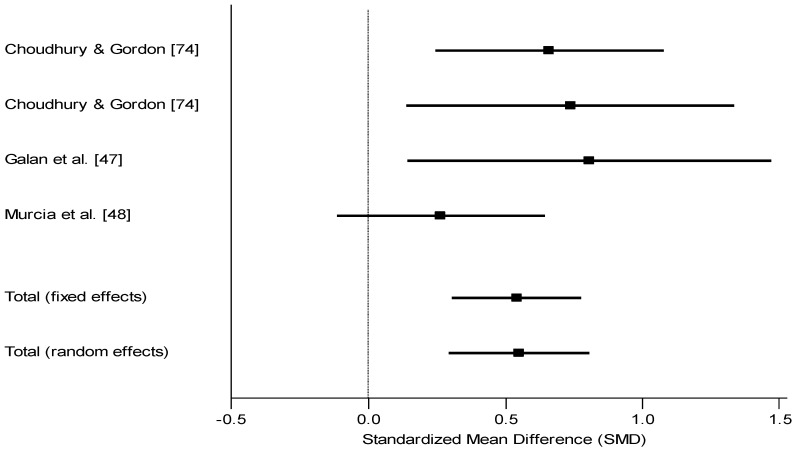
Forest plot for effect size (Standard mean difference SMD and 95% confidence interval) of iodine on mental development of children, cohort prospective studies stratified by newborn iodine status. The studies were homogeneous (*Q* = 3.44, *df* = 3, *p* > 0.05). The fixed effects model was therefore more appropriate. Estimated effect size of 0.1 in Oken’s study not included as details for computation not reported.

Exceptions to this statement include Choudhury *et al*. [75] from China, Riano Galan *et al*. [47] and Murcia *et al*. [48] from Spain, and Oken *et al*. [49] from the USA where deficient children were not treated. Using cord blood TSH to distinguish iodine-deficient *vs.* sufficient children (controls), Choudhury and coworkers [75] found that mildly deficient children of 7 months did not differ from controls (Mean 58.9 *vs.* 59.6; *ns*, *d* = 0.19) whereas severely deficient children did differ (Mean 57.5 *vs.* 59.6; *p* < 0.05, *d* = 0.66). At 13 months, using the Bayley, differences were found between controls and the severely deficient children (*d* = 0.74) [75]. Riano Galan *et al*. [47] found differences on the McCarthy measure at 40 months, again using neonatal TSH as the main indicator of iodine status (Mean for verbal 49.2 *vs.* 56.9; *p* < 0.05, *d* = 0.81; Mean for perceptual 48.6 *vs.* 54.2; *p* < 0.05, *d* = 0.64). As expected, the overall McCarthy score was also significant. Murcia *et al*. [48] did not find a difference between children grouped on the basis of their TSH (Mean 96.2 *vs.* 100.2; *ns*, *d* = 0.27). Oken *et al*. [49] found no difference between groups based on newborn T4 (because only the overall mean was provided, an effect size is estimated at between 0.00 and 0.20 = 0.10). On the basis of these four studies with five comparisons the mean effect size was 0.54 or 8.1 IQ points (Figure 4).

Five other studies, whose goal was to examine the efficacy of treatment for congenital hypothyroid children in comparison with normal controls, include Bongers-Schokking *et al*. [74], Rovet *et al*. [76,77] and Tillotson *et al*. [78]. Their findings are described here but the effect sizes were not integrated with the previous four studies. Bongers-Schokking *et al*. [74] found that severe *vs.* mild deficiency (*d* = 0.80) and early *vs.* late treatment for severe cases (*d* = 0.91) made a difference in the child’s mental development. Tillotson *et al*. [78] found no difference between affected children and controls (*d* = 0.45). Rovet *et al*. [76,77] used a number of different comparison groups, including siblings and children with non-delayed skeletal maturity as a proxy for iodine sufficiency during fetal development. Children were followed up over many years. The effects sizes for different ages and different tests ranged widely from 0.00 to 4.00. No average effect size was calculated for these different studies because it would not appropriately capture their varied outcomes.

## 4. Discussion

The findings by intervention designs, whether randomized or non-randomized, showed a consistently better outcome for children of mothers who were supplemented with iodine before or during pregnancy compared to placebo or no supplementation. The average effect size for the two randomized controlled studies was *d* = 0.68 and for the eight non-randomized studies was *d* = 0.46. The mean effect size for supplementation studies was therefore 0.49 which translates into 7.4 IQ points assuming a SD of 15. This mean effect size included a total of 16 effect sizes across 10 studies.

The findings from observation studies showed a consistently positive association between status of mother or infant and mental development. Observation studies where children’s mental development was compared on the basis of their mother’s iodine status showed a mean effect size of 0.52 which translates into 7.8 IQ points. Observational studies where children’s outcome was compared based on their own iodine status showed a mean effect size of 0.54 which translates into 8.1 IQ points. 

Several studies allowed us to examine the importance of the timing of supplementation. The findings overall showed that comparisons between early pregnancy and control groups produced a large average effect size of 0.51 while comparisons between late pregnancy and control groups produced a small average effect size of 0.17. This result was similar to two studies which included comparisons between children supplemented in early infancy *vs.* controls where differences were non-significant [57,63].

The effect size of 0.49 reported for supplementation studies was lower than reported in two other meta-analyses which combined intervention and observation designs [24,26], but only slightly lower than Verhoef *et al*. who reported an effect size of 0.56 for intervention studies [25]. Effects sizes for the other two observational designs were also similar at 0.52 and 0.54. This was slightly lower than Verhoef and coworkers’ pooled effect size of 0.67 for observational studies [25]. Higher effect sizes were reported for the two randomized design studies included the current review. However, the study by Pretell *et al*. from Peru [50] did not clearly report the procedure of randomizing women and selected post hoc for testing only the children of women supplemented prior to conception. Both studies were published before reporting guidelines became available, so a number of details such as concealment of allocation and intention-to-treat were not clearly stated. The effect sizes of this current review translate into a range of IQ points from 6.9 to 10.2 lost due to iodine deficiency, assuming that iodine treated or iodine sufficient groups attain closer to expected levels and others have “lost” IQ points. An earlier meta-analysis reported an effect size of 0.90 but this was based on observation studies of children and adults using a variety of designs and mental development measures [24]. The limitations of this meta-analysis have been mentioned earlier in this paper. The meta-analysis of studies in China used observational and non-randomized designs and the range of effect sizes was 0.58 to 0.83 depending on the design [26]. We believe that the best estimate to date of effect size in children 5 years old and under due to iodine deficiency in utero or early infancy is 0.49, which translates into 7.4 IQ points lost due to iodine deficiency. This estimate is based on the intervention studies, which despite some limitations had relatively rigorous designs and age-appropriate mental development tests.

Caution should be used when interpreting the findings from this review. Our discussion of limitations will be grouped under the following points: confounders that arise in non-randomized designs; sample size and accommodation for clustering; iodine biomarkers; conceptualization of mental development; unmeasured side effects of maternal iodine status; and consideration of the use of iodized salt on mental development.

Confounders in non-randomized and cluster randomized studies were rarely addressed. These include known correlates of mental development which might differ between intervention groups, such as family assets, family dietary diversity, mother’s education, and home stimulation, along with the weight, height and prematurity status of children [17]. Assigning villages to receive supplements also leaves open to question the potential differences among mothers in the two villages. Baseline differences in social and economic status would normally be measured and statistically controlled in the final analysis. There was no mention of such measurement or statistical control in the eight studies using non-randomized supplemented groups. Some differences in nutritional status were reported [46,58,60,61], suggesting that other unmeasured differences existed. Prospective cohort studies would be even more affected by confounds. Characteristics of the mother, such as her distance from the clinic and her years of schooling would influence children’s mental outcomes if supplementation depended on the timing of her arrival at the clinic [47,65]. Failure to include confounds in the statistical analysis may serve to inflate effect sizes. While we have addressed here only the issue of controlling for confounds within a study, there is the additional caveat concerning comparisons across studies where differences in socioeconomic status, access to health services, and nutritional status other than iodine may account for differences in mental development and may modify the effects of iodine supplementation on mental development. Sample sizes were generally small with the median at 50 per group. A properly powered study using one of the conventional measures of mental development would require a sample of 85 per group if an effect size of 0.50 was expected. The sample would need to be doubled if participants were assigned as clusters. Of the eight non-randomized studies, only two reached these numbers in the supplemented but not the control groups [57,65]. Studies assigning whole villages to receive iodine supplements did not calculate the intra-cluster correlation or accommodate clustering in their sample size estimation and statistical analysis [46,58,60,61]. Without such accommodation, effects sizes may be inflated. Another issue concerns attrition; this appears to be the case in one RCT [52,53,54,55] where only 60% of pregnant women were included in the sample because the others did not deliver at the hospital. The remaining women might have delivered at home, and their children would be expected to differ in many ways from those delivered in a hospital. 

Iodine biomarkers were measured at the baseline in most studies, though those using a cluster design often reported iodine status of the whole community, not the participating mothers. Reports of iodine status at the time of mental development assessment occurred in the two randomized studies and in those using the prospective cohort design, but rarely in non-randomized studies using supplemented groups. Information on the mothers’ and children’s iodine status would be important. For example, Berbel *et al*. [56] found that children’s iodine status was normal even when their mothers were supplemented only after delivery. Four non-randomized studies conducted in Ecuador did not assess iodine status of children or mothers. The two randomized studies did measure children’s early biomarkers and found differences due to supplementation in some such as T4, TSH and UIE. This is important because some studies using individual randomized designs with older children found contamination between groups that washed out differences [79]. Future studies might also examine maternal thyroid antibodies as it has been suggested that they are a marker of thyroid dysfunction [38].

Fortunately most of the studies in this review used conventional measures of mental development, such as the Brunet-Lézine, Bayley, and Stanford-Binet. These scales require modification to adapt to the local context because asking young children about objects they have never seen would not validly assess their competence. A recent cross-cultural longitudinal study of infants 3- to 9-months old from Cameroon and Germany also supports the importance of checking on the correct sequence of items so that they reflect context-specific levels of difficulty [80]. Most of the researchers in the current review reported making modifications, but we have not been able to assess the appropriateness which would require local knowledge or validation in the setting. It would not be possible to normalize the scores as this would require a national representative sample. Instead, we assume that they standardized scores based on the internationally available norms. Although the standardized score may not be valid for the country, all children in the sample were subject to the same standard. The median score for such measures is expected to be 100. Most of these children would be expected to score considerably lower if they had iodine deficiency. This was the case for the study by Pretell *et al*. in Peru [50] where the unsupplemented children had a mean score of 75.0. In contrast, Thilly’s study in the DR Congo [52,53,54,55] produced a mean of 103 for the placebo group, still well below the supplemented group with a mean of 115. These high scores among iodine-deficient children suggest they have normal or slightly above-average intelligence in this context or that the modifications made the test easier. A major shortcoming for many of the studies was their failure to provide separate scores for language and cognitive (mainly non-verbal) outcomes. All the measures used allowed for such a distinction and developmental scientists consider this to be an important distinction. Some iodine supplementation studies among older children have analyzed language and non-verbal cognitive scores separately and found that cognitive outcomes are more likely to show group differences than language outcomes [15,81,82], although others found no group differences for either language or cognitive outcomes (e.g., [19,83]), and some found an inconsistent mix [21]. Some researchers measured only fine motor skills such as putting pegs in the holes of a pegboard and threading beads [84]; this is a non-verbal item in the Bayley I measure for which cognitive and fine motor skills were combined into one score. In the Bayley III version [85], cognitive skills are scored separately from fine motor. Gross motor skills (measured by Pharoah *et al*. [35] in New Guinea) such as sitting, crawling, and walking are not considered to be mental development outcomes and so were not included in this review. They are not clearly related to mental development outcomes unless so severe as to be considered a sign of cretinism. In brief, it would be useful for researchers to analyze separately the language and cognitive subscale scores to map differences on to the findings of older children.

Unmeasured positive consequences of iodine supplements given to mothers were not considered in any of the articles. These include other changes in mothers that might affect mental outcomes in their children. Mothers with better iodine status, and therefore thyroid function, might be more energetic and less depressed [86,87]. This may in turn lead to more positive and stimulating interactions with their infants. In most low-income and middle-income countries, children receive low levels of stimulation via conversation and play materials [17]. Use of the HOME Inventory worldwide has now established that low levels of stimulation strongly predict low levels of mental development in children [88]. Likewise, in some regions mothers low in positive emotion tend to provide poorer diets and stimulation to their children resulting in lower weight, height, and mental development [89,90]. Because none of the reviewed studies examined mothers’ parenting practices after delivery, we cannot determine how much of the difference is explained by the child’s iodine sufficiency and how much is explained by the mother’s provision of positive stimulation rather than the child’s iodine sufficiency. 

Finally, only two studies reported here addressed levels of iodized salt in the diets of mothers [47,48]. It is assumed that the studies providing supplementation were in areas with little or no access to iodized salt. Supplementation was in the form of an injection or capsule which provided enough iodine for a year or more. However, capsules are a short-term intervention, not a long term solution to the problem of endemic iodine deficiency. In contrast to studies reported here and other meta-analyses, the meta-analysis of 37 studies from China mainly compared samples that differed in their access to iodized salt [26]. Universal salt iodization is recommended by WHO and UNICEF for the sustainable elimination of iodine deficiency in all countries where iodine deficiency disorders are of public health concern [91]. Despite the success of salt iodization programs in many countries around the world, some countries still have low coverage of adequately iodized salt [92]. In settings where a large proportion of the population does not have access to adequately iodized salt, WHO and UNICEF recommend high dose iodine supplementation for the most vulnerable groups as an interim measure until salt iodization can be scaled up. Additional research and recommendations suggest the need to complement iodized salt, when it has been used for a short time only or when of low quality, with iodine supplementation in women of child-bearing age [93,94]. To determine if iodized salt in the maternal or child diet has the same effects on mental development as high dose supplementation, effectiveness research will have to take place in these countries.

## 5. Conclusions

Our review found that regardless of study design, iodine deficiency had a biologically important impact on, or association with, mental development. We believe that the best estimate to date of the effect size of iodine supplementation on mental development in children 5 years old and under is 0.49, which translates into 7.4 IQ points lost due to iodine deficiency. Although these intervention studies had relatively rigorous designs and used age-appropriate mental development tests, results must be interpreted with caution due to various limitations in study design and analysis including small sample size and possible attrition, lack of accommodation for intra-cluster correlation, and inattention to confounding variables. The observational studies, albeit a weak design, support the idea that iodine is important for mental development, and provide similar effects sizes as the intervention studies. To further quantify the importance of iodine for mental development in young children, this review points to the need for well-designed randomized controlled trials with adequate sample sizes as well as studies that assess the effectiveness of iodized salt.
