# Correction: Bougma, K., *et al.* Iodine and Mental Development of Children 5 Years Old and Under: A Systematic Review and Meta-Analysis. *Nutrients* 2013, *5*, 1384–1416

**DOI:** 10.3390/nu6125770

**Published:** 2014-12-10

**Authors:** Karim Bougma, Frances E. Aboud, Kimberly B. Harding, Grace S. Marquis

**Affiliations:** 1 School of Dietetics and Human Nutrition, McGill University, 21111 Lakeshore Road, CINE Building, Sainte Anne-de-Bellevue, QC, H9X 3V9, Canada; E-Mail: karim.bougma@mail.mcgill.ca; 2 Department of Psychology, McGill University, 1205 Dr. Penfield Avenue, Montreal, QC, H3A 1B1, Canada; E-Mail: frances.aboud@mcgill.ca; 3 Micronutrient Initiative, 180 Elgin Street, Suite 1000, Ottawa, ON, K2P 2K3, Canada; E-Mail: kharding@micronutrient.org

We have found an inadvertent error in our paper published in *Nutrients* [1]. One standard error was not converted into standard deviation and this affected the following effect sizes and intelligence quotient (IQ):

On page 1384, the sentences “Average effect sizes for these four designs were ***0.68*** (2 RCT studies), 0.46 (8 non-RCT studies), 0.52 (9 cohort stratified by mothers’ iodine status), and 0.54 (4 cohort stratified by infants’ iodine status). This translates into 6.9 to ***10.2*** IQ points lower in iodine deficient children compared with iodine replete children” should be “Average effect sizes for these four designs were ***0.48*** (2 RCT studies), 0.46 (8 non-RCT studies), 0.52 (9 cohort stratified by mothers’ iodine status), and 0.54 (4 cohort stratified by infants’ iodine status). This translates into 6.9 to ***8.1*** IQ points lower in iodine deficient children compared with iodine replete children”. 

On page 1391, Table 1, “G1 (115 ± ***3***) > Gc (103 ± ***4***) **; ***0.99 (0.50, 1.48)***” should be “G1 (115 ± ***18.7***) > Gc (103 ± ***24.0***) **; ***0.56 (0.09, 1.02)***”.

On page 1400, the sentences “Thilly *et al.* [52–55] found that supplementation during the second and third trimester with a single dose of 475 mg of iodine resulted in a statistically significant difference in children’s mental outcome compared to the placebo control group (Mean 115 *vs.* 103; *p* < 0.01, d = ***0.99***). The average effect size from these two double blind RCT was d = ***0.68***.” should be “Thilly *et al.* [52–55] found that supplementation during the second and third trimester with a single dose of 475 mg of iodine resulted in a statistically significant difference in children’s mental outcome compared to the placebo control group (Mean 115 *vs.* 103; *p* < 0.01, d = ***0.56***). The average effect size from these two double blind RCT was d = ***0.48***”.

On page 1401, the sentence “The average effect size for randomized (d = ***0.68***) and non-randomized (d = 0.46) designs was ***0.49***.” should be “The average effect size for randomized (d = ***0.48***) and non-randomized (d = 0.46) designs was ***0.50***.”

On page 1402, the sentence “Consequently, an effect size of ***0.49*** translates into a ***7.4*** IQ point difference (***0.49*** × 15) between groups.” should be “Consequently, an effect size of ***0.50*** translates into a ***7.5*** IQ point difference (***0.50*** × 15) between groups.”

On page 1402, Figure 2 title, “(Q = ***54.81***, df = 15, *p* < 0.0001)” should be “(Q = ***54.57***, df = 15, *p* < 0.0001)”.

On page 1405, the sentence “The average effect size for the two randomized controlled studies was d = ***0.68*** and for the eight non-randomized studies was d = 0.46” should be “The average effect size for the two randomized controlled studies was d = ***0.48*** and for the eight non-randomized studies was d = 0.46”.

On page 1406, the sentence “The mean effect size for supplementation studies was therefore ***0.49*** which translates into ***7.4*** IQ points assuming a SD of 15” should be “The mean effect size for supplementation studies was therefore ***0.50*** which translates into ***7.5*** IQ points assuming a SD of 15”.

On page 1409, the sentence “We believe that the best estimate to date of the effect size of iodine supplementation on mental development in children 5 years old and under is ***0.49***, which translates into ***7.4*** IQ points lost due to iodine deficiency.” Should be “We believe that the best estimate to date of the effect size of iodine supplementation on mental development in children 5 years old and under is ***0.50***, which translates into ***7.5*** IQ points lost due to iodine deficiency.”

These changes have no material impact on the conclusions of our paper. We apologize to our readers.
